# Interdependence of Rheological and Biochemical Parameters of Blood in a Group of Patients with Clinically Silent Multifocal Vascular Cerebral Lesions

**DOI:** 10.3390/biomedicines11072063

**Published:** 2023-07-22

**Authors:** Anna Marcinkowska-Gapińska, Izabela Siemieniak, Weronika Kawałkiewicz, Olgierd Stieler, Dorota Hojan-Jezierska, Leszek Kubisz

**Affiliations:** 1 Department of Biophysics, Poznan University of Medical Sciences, 61-701 Poznań, Poland; wkawalkiewicz@ump.edu.pl (W.K.); lkubisz@ump.edu.pl (L.K.); 2 Rheological Laboratory, Department of Neurology, Poznan University of Medical Sciences, 61-701 Poznań, Poland; 3 Department of Hearing Healthcare Profession, Poznan University of Medical Sciences, 61-701 Poznań, Poland; osa@ump.edu.pl (O.S.); djeziers@ump.edu.pl (D.H.-J.)

**Keywords:** hemorheology, blood viscosity, cerebrovascular disease, cerebral ischemia

## Abstract

Background: Hemorheology is a field of science which often becomes interesting to researchers studying impairments related to blood flow disturbances. Clinically silent vascular cerebral lesions (CSVCLs) are considered a problem of great importance in neurology. Objective: This work aimed to analyze the interdependencies of the rheological and biochemical parameters of the blood. Methods: The group of patients included persons with clinically silent multifocal vascular cerebral lesions diagnosed using neuroimaging. The control group had no symptoms in the central nervous system (CNS). We analyzed hemorheological profiles in 69 patients with CSVCLs diagnosed via magnetic resonance imaging (MR) or 64-row computer tomography measurements. Rheological data were acquired using a rotary-oscillating rheometer, the Contraves LS-40, an instrument dedicated to blood viscosity measurements. For each sample, the hematocrit value was measured using the standard method. Analysis of erythrocytes’ aggregability and deformability was performed using the rheological model of Quemada. Biochemical tests of blood were also performed. Results: The results of rheological and biochemical studies were compared with those obtained in the control group. Special attention was paid to the correlation analysis of rheological and biochemical parameters. Conclusions: Such correlations were found, e.g., between the red cells’ deformability and the fibrinogen level. The results improve our understanding of blood flow hemodynamics by analyzing the shear-dependent behavior of the aggregation and deformability of red blood cells.

## 1. Introduction

The incidence of clinically silent cerebral ischemic lesions (CSVCLs) is surprisingly high. Based on the results of neuroimaging studies (CT and NMR), it is estimated that in the general population of healthy people without a history of TIA or ischemic stroke, silent foci of vascular brain injury (SBI) may occur in almost 50% of cases. In those who live to be over 70 years of age, SBI occurs in more than 25% of cases [1,2,3,4]. The definition of SBI results from the fact that their clinical manifestation is not related to the acute symptoms of stroke and transient cerebral ischemia. However, subtle neurological deficits can be observed, such as visual field disorders, cognitive function disorders, the deterioration of gait, the deterioration of general physical fitness, and a lowered mood or depression [2]. The incidence of clinically silent cerebral ischemic changes increases with age and may be a phenomenon accompanying the ageing process [1,2]. Based on the analyses carried out so far, it is estimated that the risk factors for SBI are consistent with the risk factors for cerebrovascular diseases, of which the most important are age, hypertension, diabetes, atrial fibrillation, elevated cholesterol and homocysteine levels, the presence of atherosclerotic plaques in the carotid arteries, and smoking [2]. Disturbances in blood flow at the level of the cerebral microcirculation lead to local hypoxia and the formation of small-sized lesions that are ischemic brain lesions of a clinically silent nature [1]. As in the case of lacunar strokes, the etiopathogenesis of this silent insufficiency in the brain is caused by cerebral small vessel disease, mainly a cerebral complication of arterial hypertension [2,3]. However, the mechanisms leading to microcirculatory flow disturbances are not fully understood [1].

The branch of science that deals with the study of the flow of liquids and the phenomena accompanying this flow is rheology, which is related to blood hemorheology. The history of hemorheological studies has shown that changes in the rheological properties of blood are associated with the pathogenesis of many cardiovascular, hematological, metabolic, inflammatory, autoimmune and endocrine diseases [5,6,7]. As more and more knowledge exists about the processes involved in blood flow, it has become clear that individual parameters behave differently depending on the underlying disease. Many published studies on the acute phase of ischemic stroke have shown increased blood viscosity compared to healthy controls [8,9,10,11]. The increase in the concentration of fibrinogen, a protein involved in forming erythrocyte aggregates that significantly affect the value of plasma viscosity and, thus, the viscosity of whole blood [9,12,13], partly explains the situation. In the case of chronic cerebral ischemia, researchers indicate an increased viscosity of whole blood and plasma but also an increase in the hematocrit value and the ability of erythrocytes to aggregate [8,9,10,11,14]. Another important rheological parameter is the ability of erythrocytes to deform. Studies indicate that this parameter remains unchanged or decreases in the acute phase of ischemic stroke, while in the chronic phase, it decreases [8,9,10,11,13] or increases [9]. It remains an open question as to whether changes in hemorheological parameters are significant for the development of cerebral ischemia, including clinically silent cerebral vascular lesions (CSVCLs), and if so, what these changes are and whether they are a causative factor, or do they only accompany ischemic processes.

Deviations in blood rheological parameters are found in over 40% of people with cerebral ischemia [13]. The metabolic activity of the brain is very high, which is made possible by the intense local blood flow. Hemorheological imbalance in the cerebral microcirculation may lead to ischemic strokes and other disorders, such as transient ischemic attack and the increasingly diagnosed silent cerebral infarction (SBI) [10,14]. Nerve cells do not have the capacity for anaerobic metabolism; hence, their activity without strictly being conditioned by the uninterrupted supply of oxygen and glucose is impossible. The blood flowing through both large vessels and microcirculation provides a continuous supply of these vitally important substances to the brain [8]. Analysis of the mechanisms of circulatory disorders in a group of patients with completed ischemic stroke, transient cerebral ischemia, lacunar infarction and cerebral ischemic foci is critical. Due to the similarity of risk factors between patients with silent cerebral ischemia foci and patients with ischemic stroke, the data of patients with cerebral ischemia were analyzed regarding the number of ischemic foci shown in the NMR examination. These studies showed that patients in the high-stage group (>3) had higher fibrinogen levels than those in the low-stage group (0–3), but in both cases, these levels were lower than that in patients with chronic cerebral ischemia [10]. Despite many analyses of the mutual correlations of individual hemorrhagic parameters in cerebrovascular diseases, the mechanisms of the occurrence of disorders have not been fully explained [8,9,10,11,13]. It is also unknown as to what factors determine the occurrence of clinically silent cerebrovascular lesions (CSVCLs) [1].

Mechanisms of blood circulation disorders are a subject of interest in the study of hemodynamics [5,6,7,15,16,17]. Hemorheology also includes the analysis of blood flow abnormalities related to the changes in blood biochemical parameters in various diseases and other conditions, such as pregnancy [5,6,7,15,16,17,18]. The Fundamentals of knowledge of hemorheological parameters are increasingly used to analyze the causes and development of vascular diseases. For example, model parameters of a non-Newtonian fluid representing blood can be interpreted in terms of the rheological properties of plasma and the blood morphological components [19,20]. Hemorheological studies focus primarily on measuring blood viscosity as a function of shear rate, plasma viscosity, hematocrit (Hct) and red cells’ aggregability and deformability. For example, whole-blood viscosity may change from about 4 mPas at high shear rates (*γ*′, above 100 s^−1^) to about 100 mPas at low shear rates (below 1 s^−1^) [5,6]. The phenomena of deformation and aggregation are significant for maintaining blood fluidity. In the range of low shear rates, in physiological conditions close to stagnation, the dominant phenomenon is the aggregation of erythrocytes. In contrast, in the range of high shear rates (small vessels), the deformation of erythrocytes dominates [5,6]. The viscosity of whole blood also varies depending on the composition of the plasma, the viscosity of which is significantly influenced by the presence of macromolecular proteins, especially fibrinogen [12]. Blood viscosity has been studied in many research centers and among many different groups of patients. The main disorders related to the hemorheological properties are coronary insufficiency, vascular congestion, myocardial infarction, a cerebral circulation disorder, Reynaud disease, ischemic limbs, diabetes, anemia, and tumors [5,7,8,15,16,21].

Analyzing the influence of hemorheological parameters on the formation of silent ischemic lesions of the brain in patients with clinically silent ischemic episodes of the brain detected by means of neuroimaging (CT, MRI) is crucial to expand the knowledge on the role of rheological blood disorders in the morbid process’ etiology.

This study aimed to find correlations between the hemorheological and biochemical parameters in two groups of patients: one with clinically silent multifocal vascular cerebral lesions and a reference group consisting of patients suffering from similar diseases but without visible ischemic foci in neuroimaging diagnosis.

## 2. Materials and Methods

### 2.1. Criteria for the Choice of Patients and Group Assignment

We analyzed the hemorheological profile of 69 patients with CSVCL diagnosed via magnetic resonance imaging (MRI) or 64-row computed tomography (CT) compared to the control group of 17 subjects without such changes. The control group consisted of patients on the same ward hospitalized due to similar problems related to the nervous system, but without any ischemic lesions of the brain, as confirmed by neurological and neuroimaging examination. The test group included neurological ward patients with CSVCL who volunteered to donate blood. All of them suffered from ailments affecting the nervous system, and many of them were at risk for cerebrovascular diseases. Twenty-one patients at the time of admission to the ward were subjected to statin therapy. Additionally, depending on the underlying disease, patients took drugs for hypotension and hypoglycemia, nootropics and psychotropics and drugs reducing blood clotting. The number of smokers in both groups was proportional.

Patients who met the following criteria were included in this study:

- No history of ischemic stroke or TIA
- Neurological examination revealed only subtle deficits in physical and cognitive functioning [8]. Each patient had their neurological status monitored several times.
- Neuroimaging examinations performed in patients revealed the presence of foci meeting the criteria for ischemic lesions: in MRI images taken in the T1, T2 and FLAIR sequences, the ischemic lesion should not be smaller than 3 mm in diameter and must be hyperintense in the T2 and FLAIR sequences (differentiation from perivascular spaces of Wirchow-Robin). In 64-slice CT, the assessment of scans was performed for the presence of hypodense foci (post-ischemic glial scars). The changes in the brain found in the studies mentioned above met the radiological criteria for ischemic changes.
- The third criterion was used to differentiate the patients between the main and the reference group.

Patients with a history of ischemic stroke and transient cerebral ischemia were excluded from the study group; persons living with the patient supplemented the interview.

### 2.2. Demographic Data

Table 1 and Table 2 present the demographic characteristics of the study groups.

The groups did not differ in age and comorbidities, and so the most important differentiating factor was the presence of clinically silent ischemic foci in the neuroimaging examination. Patients in the treatment and control groups were burdened with risk factors for cerebrovascular diseases. Table 3 shows the load-associated diseases for the cases of patients and control subjects.

### 2.3. CSVCL Diagnosis

All MRI scans were performed on a Siemens Magnetom Vision 1.5 T (Siemens, Munich, Germany) whole-body MR scanner. T1- and T2-weighted imaging and fluid-attenuated inversion recovery (FLAIR) were performed. The computed tomography (CT) scans were performed on a Siemens Somatom Sensation 64 instrument (Siemens, Munich, Germany). A radiologist blinded to the clinical data analyzed the results in both cases.

### 2.4. Biochemical Parameters

All patients were routinely subjected to tests measuring the blood count, blood sugar, urea, creatine serum, lipid profile, cholesterol, fibrinogen, IgM, IgG, IgA, and albumin/globulin ratio (A/G), along with thyroid tests, liver tests, urinalysis and chest X-rays. All patients in both groups underwent several tests of their neurological condition.

### 2.5. Blood Collection and Rheology

Blood for hemorheological tests was collected using the regular procedures (for example, morning hours, empty stomach). Before blood sampling, the patient was calm. Blood collection was performed with a needle inserted into a vein feeding directly into a sterile tube with an anticoagulant, exerting minimal pressure on the limb. Blood was collected in Vacutest^®^ tubes (4 mL, with K_3_EDTA). The time from collection to measurement did not exceed four hours to avoid the necessity of any special treatment of blood samples [22]. All viscosity measurements were performed using a rotary-oscillating rheometer Contraves LS40 (Zurich, Switzerland) at 37 °C. The measurement system used is the Couette DIN 412 system, whose geometric parameters according to the specification are: internal radius (Ri) 6 mm; external radius (Ra) 6.5 mm; length of the contact surface of the sample (L) 18 mm; and volume of the sample, 1.8 mL. Whole-blood viscosity was measured in the order of decreasing shear rate in the range of 100–0.01 s^−1^ within 5 min after short pre-shearing at *γ*′ = 100 s^−1^. Plasma viscosity was estimated from a regression analysis of the shear rate dependence of shear stress measured in the range of *γ*′ = 10–100 s^−1^, optimal for a Newtonian liquid of such low viscosity. Each blood sample’s hematocrit value was measured using the standard method (centrifuging the sample for 10 min at 4000 rpm).

The following hemorheological parameters were estimated: relative blood viscosity at various shear rates, plasma viscosity, hematocrit, and parameters of Quemada’s rheological model (with *k_∞_* as red cell deformability and *k*_0_ and *γ*′_c_ as red cell aggregation). Equations (1) and (2) show the formula of Quemada’s rheological model [6,23,24]

(1) $$\eta \left(\gamma \prime \right)=\frac{\tau \left(\gamma \prime \right)}{\gamma \prime }=\eta_{p}{\left[1-\frac{1}{2}k_{Q}\cdot Hct\right]}^{-2}$$

(2) $$k_{Q}=\frac{k_{0}+k_{\infty }\sqrt{\gamma \prime /\gamma \prime_{c}}}{1+\sqrt{\gamma \prime /\gamma \prime_{c}}}$$

### 2.6. Fitting Routine and Statistical Parameters

The parameters of Quemada’s model were obtained by fitting the model (Equations (1) and (2)) to the experimental flow curve using a non-linear fitting procedure based on the Levenberg–Marquardt algorithm published in “Numerical recipes” [25]. A self-written program (Delphi, Borland, USA) was used to speed up the data treatment, including reading raw data files, their fitting with various models, adding biochemical and other parameters and performing statistical and correlation analysis. The Shapiro–Wilk method was used to check the normality of the distribution of all parameters in respective groups. The value of 5% was adopted as the criterion for the significance level. The significance of differences in mean values between the study groups was checked for the studied parameters using Student’s *t*-test.

## 3. Results

**Figure 1:**
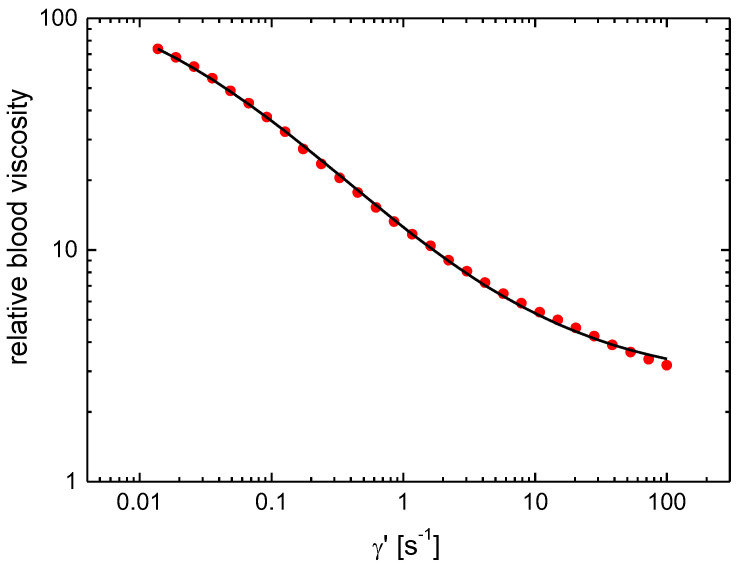
A typical plot of relative blood viscosity as a function of the shear rate in the 0.01 to 100 s^−1^ range. The points on the graph represent the experimental data of one of the patients, and the solid line fits the mathematical Quemada model.

Table 4 presents the results of the measurements in the group of patients and the control group. The hematocrit values were measured in all blood samples, and the first row lists their mean values and the standard deviations of the mean value. The second row contains mean plasma viscosity values for each group with their standard deviations of the mean value. The next four rows show whole-blood viscosity values measured at four chosen shear rates listed in the table. The end of Table 4 lists the Quemada rheological model parameters values *k*_0_, *k*_∞_ and *γ*′_c_ calculated from the flow curves using a non-linear fitting procedure. Figure 1 shows a typical plot of measured relative blood viscosity as a function of the shear rate (points) and the Quemada model fit (line) taken from one of the tested blood samples.

The biochemical parameters were measured for each patient to enrich the interpretation of the rheological properties in patients with clinically silent brain ischemic foci. Table 5 shows their average values. We found significant differences between the control group and the group of patients. We observed a significant increase in red cell elasticity *k_∞_* (*p* < 0.04) and IgG level (*p* < 0.05) in the patient group as well as a significant decrease in fibrinogen level (*p* < 0.047), IgM level (*p* < 0.001), and A/G ratio (*p* < 0.015).

**Figure 2:**
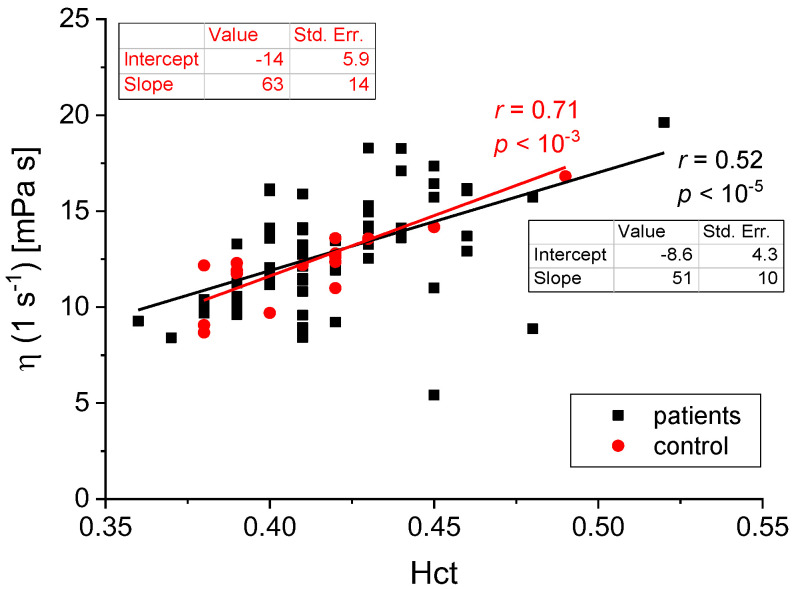
The correlation between the patients and the control group is between *η*(1 s−^1^) and Hct.

**Figure 3:**
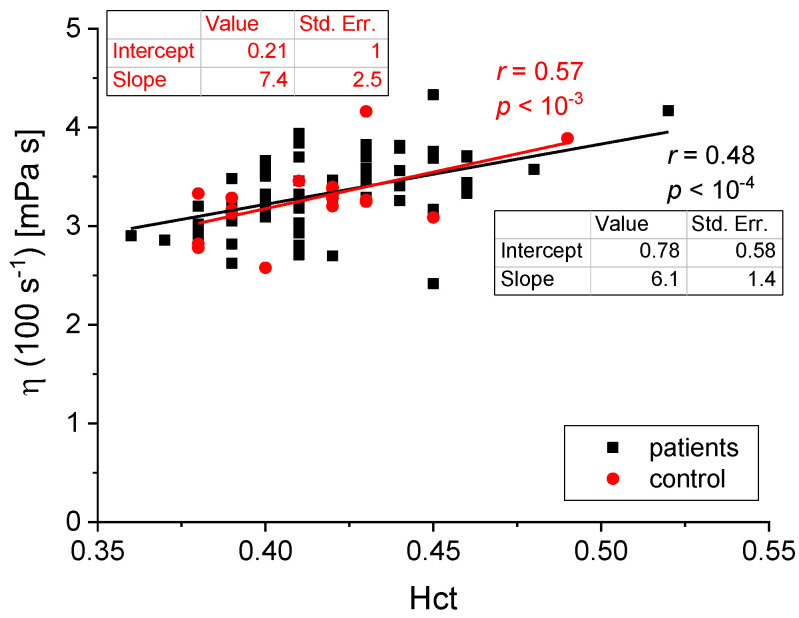
Correlation between *η*(100 s^−1^) and Hct for the patients and the control group.

**Figure 4:**
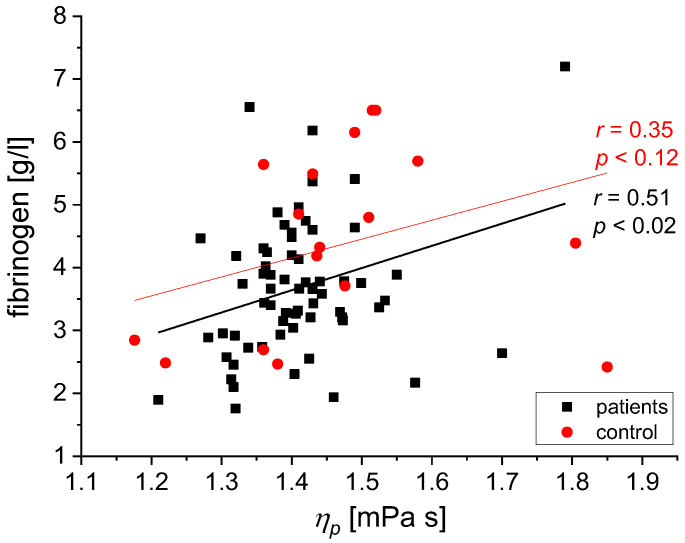
Correlation between fibrinogen level and *η*_p_ for the patients and the control group.

**Figure 5:**
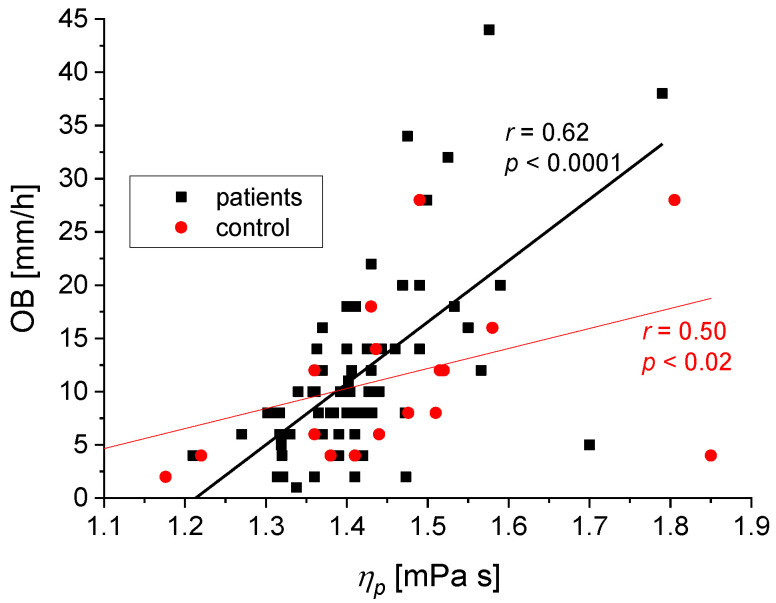
Correlation between the OB value and *η*_p_ for the patients and the control group. The two slopes (58 and 19 mm/(h mPas)) are significantly different (*p* = 0.05).

**Figure 6:**
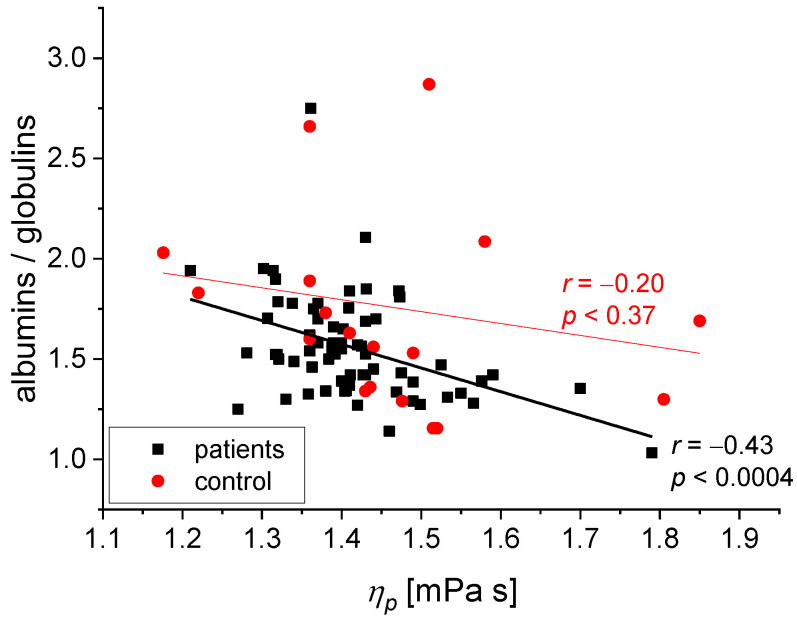
Correlation between the A/G ratio and *η*_p_ for the patients and the control group.

**Figure 7:**
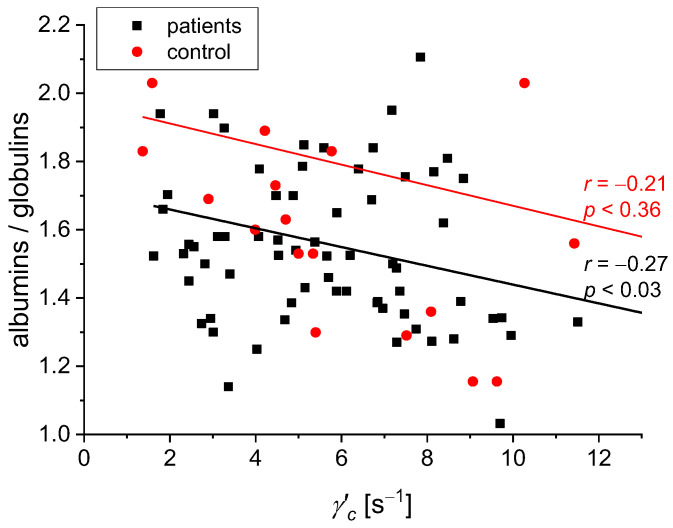
Correlation between the A/G ratio and *γ′*_c_ for the patients and the control group.

**Figure 8:**
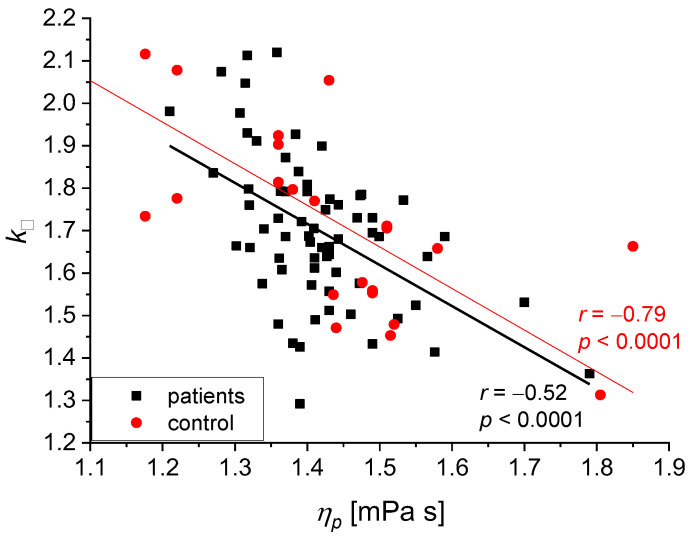
Correlation between the *k_∞_* parameter and *η*_p_ for the patients and the control group. The two slopes are not different (*p* = 0.02).

**Figure 9:**
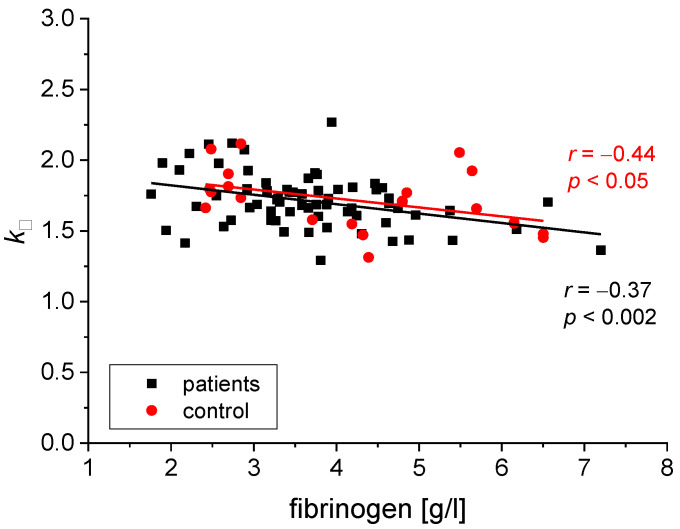
Correlation between the *k_∞_* parameter and the fibrinogen level for the patients and the control group. The two slopes are not different (*p* = 0.05).

**Figure 10:**
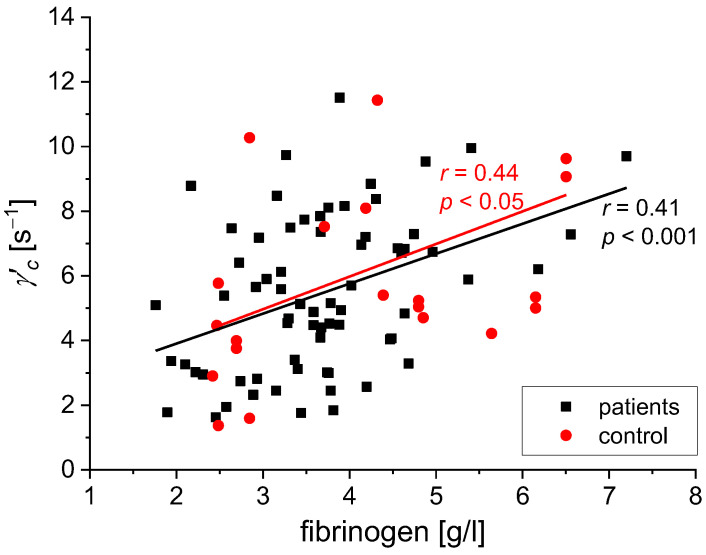
Correlation between the *γ′*_c_ parameter and the fibrinogen level for the patients and the control group. The two slopes are different (*p* = 0.11).

We found the following statistically significant correlations between the rheological and biochemical parameters: relative blood viscosity and hematocrit value (Figure 2 and Figure 3), fibrinogen and plasma viscosity (*p* < 0.02 in patients) (Figure 4), OB (ESR) and plasma viscosity (*η*_p_) (*p* < 0.0001 in patients) (Figure 5), A/G and plasma viscosity (*η*_p_) (negative in patients, *p* < 0.0004) (Figure 6), A/G and *γ′*_c_ (RCA) (*p* < 0.03 in patients) (Figure 7), a parameter of the Quemada model *k_∞_* and plasma viscosity (*η*_p_) (*p* < 0.0001) (Figure 8), a parameter of the Quemada model *k_∞_* and fibrinogen (*p* < 0.002 in patients) (Figure 9), and a parameter of the Quemada model *γ′*_c_ and fibrinogen (*p* < 0.05 and in patients, *p* < 0.001) (Figure 10).

## 4. Discussion

Hemorheological studies are usually based on the evaluation of blood fluidity in vitro. Parameters of special interest are red cell deformability and ability to shift orientation in flow, aggregability, whole-blood viscosity and blood plasma viscosity. Mechanisms of blood flow disorders in vascular diseases of the brain, particularly in clinically silent ischemic lesions of the brain, remain unclear, despite the many studies performed so far [14]. The formation of these changes is associated with impaired circulation at the capillary level, where keeping the balance of hemorheological parameters is a prerequisite for maintaining the flow rate. In this study, hemorheological tests were carried out in patients with confirmed clinically silent ischemic foci of the brain and in the control group where such lesions were not found. Due to the interrelation between the parameters characterizing the biochemical and rheological properties of the blood, a large set of biochemical parameters in both groups were measured in order to obtain a more comprehensive picture of the mutual relationship between the deviations found in hemorheological studies and the values of some biochemical parameters.

Table 4 summarizes the results of hemorheological parameters for the group of patients and controls. It can be noted that the Quemada model parameter *k_∞_* shows in patients a statistically significantly lower value (*p* < 0.04). The value of *k_∞_* is interpreted as an indicator of the ability of red blood cells to deform. The lower value of this parameter indicates the improvement of erythrocyte deformability. The reduced value of this indicator in patients with silent cerebral ischemic foci might suggest greater flexibility of erythrocytes in these patients in relation to the control group. The deformation phenomenon plays a special role in maintaining blood flow in the microcirculation. The underlying pathogenesis of silent ischemic lesions of the brain and lacunar stroke lies in the circulatory disorders in the capillaries, where a crucial role is played by increased blood viscosity. The results obtained in this study (Table 4) indicate that no impaired red cell deformability was observed in the patient group. The literature reports that insufficient red cell deformability may lead to circulatory disorders at the capillary level, local hypoxia of neuronal tissue or foci formation of silent ischemia [1,2,3,4,26,27]. The reverse trend in the obtained data can be attributed to other disorders found in both groups: high blood pressure, diabetes, and coronary heart disease. They all influence erythrocyte deformability to some extent [28,29,30]. On the other hand, some studies report that improved red cell flexibility for patients after remote ischemic stroke may result from adaptive mechanisms triggered by this phenomenon [9].

Studies on the rheological properties of blood claim that large plasma proteins like globulins, including fibrinogen and IgM, raise blood viscosity by increasing plasma viscosity and the aggregation rate of red blood cells. IgG molecules, although belonging to the globulins, due to their smaller size, influence blood viscosity much less. Similarly, albumins, as low-molecular-weight proteins, play a less significant role in the rheological properties of blood. Blood viscosity at a low shear rate increases with the increasing rate of the process of red blood cell packet formation, which requires the presence of fibrinogen and IgM molecules forming inter-erythrocyte bridges [28,31,32,33,34]. Based on the analysis of test results of biochemical parameters that potentially affect the rheological properties of blood (Table 5), one can conclude that in the group of patients, the values of the fibrinogen level, IgM level and the ratio of albumin/globulin are lower (*p* < 0.047, *p* < 0.001, *p* < 0.015, respectively. In contrast, the value of the IgG level is higher (*p* < 0.05). Lower levels of fibrinogen and IgG in the blood in patients (Table 5) suggest a lower tendency of erythrocytes to create packets and a reduced plasma viscosity in the microcirculation. Such observations conclude that high-molecular-weight plasma proteins do not induce the mechanism of creating foci of ischemic brain hemorheological disorders in microcirculation.

This study looked for a correlation between blood’s rheological and biochemical parameters. One of the expected relationships was that between the blood viscosity *η* measured at four selected shear rates (γ′ = 0.1, 1, 10 and 100 s^−1^) and the hematocrit value Hct in the group of patients and the control group. The plots of *η*(Hct) for γ′ = 1 s^−1^ and γ′ = 100 s^−1^ are shown in Figure 2 and Figure 3, respectively, together with the trend lines and the *p* values. This correlation corroborates the well-known effect of hematocrit on blood viscosity [5,6,7,15]. However, the key information is the difference in the slope of the curves in high- and low-shear-rate conditions. Blood viscosity changes by ~30% (trend line) in the range of high shear rates, while for low shear rates, where erythrocyte aggregation is the dominant phenomenon, blood viscosity changes almost 2-fold in the same range of hematocrit values. Such a result illustrates the significance of red cells’ mechanical and aggregation properties in the rheological behavior of whole blood. Similar plots could be made for various parameter values describing erythrocytes’ mechanical properties. However, these are not as easily accessible experimentally. Like Quemada’s model, the idea behind rheological models lies in finding a functional viscosity description based on all-important parameters. Quemada’s model includes hematocrit, shear rate, and the processes ruling the red cells’ behavior, all in a single equation. Instead of analyzing multiple dependencies of whole-blood viscosity on various parameters, like those in Figure 2 and Figure 3, for each blood sample, a complete set of parameters is obtained by fitting their values to the measured flow curve. Further analysis refers to the values of those physically meaningful parameters rather than blood viscosity, which reflect their combination’s effect.

Based on the reports of other researchers on the influence of plasma proteins, especially those of high molecular weight, on the viscosity of whole blood, it was checked whether this relationship also occurs in the study groups. Figure 4 shows that the positive correlation between fibrinogen values and plasma viscosity in the study group is consistent with previous reports [28,33,35,36], indicating that a rise in fibrinogen levels increases blood viscosity by increasing plasma viscosity [12]. Figure 5 shows a positive correlation between the values of precipitation OB (OB—Biernacki Reaction) and plasma viscosity *η*_p_. The value of the ESR parameter (ESR—erythrocyte sedimentation rate) partially correlates with the aggregation of erythrocytes in blood vessels, and in medical practice, it is interpreted as the ex vivo erythrocyte sedimentation rate [36,37]. The obtained results indicate a strong positive correlation between ESR (ESR) values of the sedimentation rate of red blood cells and plasma viscosity, especially in patients with silent non-degenerative brain lesions (*p* < 0.0001), as well as in the control group (*p* < 0.02). Clinically, elevated ESR (ESR) is associated with increased blood fibrinogen levels, elevated the immunoglobulin M, and very high values of the immunoglobulins G and A [36]. On this basis, it can be concluded that increased aggregation and increased plasma viscosity may play an important role in the pathogenesis of silent cerebral ischemic lesions.

A negative correlation between the ratio of albumin/globulin and plasma viscosity was found in the patient group (Figure 6). Based on reports in the literature [32], a reduced value of the ratio of albumin/globulin is an indicator of an increased risk of vascular events. A low albumin/globulin ratio value may result from decreased albumin concentration or higher globulin content. The value of the ratio of albumin/globulin is inversely proportional to the concentration of the globulins, including fibrinogen, in the serum. The relationship between the albumin/globulin ratio and *η*_p_ shown in Figure 6 corroborates the literature data [38]. This confirms that the increase in plasma viscosity is affected by the profile of serum proteins: a low content of albumins and a high level of globulins and fibrinogen. Silent cerebral ischemic lesions are the result of disturbances in the microcirculation of blood flow: in the capillaries with a diameter below 5–7 microns, a reversal of the Fahraeus–Lindqvist effect occurs, leading to an increase in blood viscosity [5,32,39,40]. Based on the correlation shown in Figure 6, we can conclude that in the group of patients, low plasma viscosity values correspond with a higher ratio of albumin/globulin and a lower content of fibrinogen and gamma globulins in serum. Given these facts, we can state that the plasma proteins do not affect the creation of conditions hindering the blood flow in the microcirculation, essential in forming silent ischemic lesions of the brain.

Another correlation test was performed for the ratio of albumin/globulin and the γ′_c_ value (Figure 7). In the test group, we observed a negative correlation (*p* < 0.03) between the ratio of albumin/globulin and the parameter of Quemada’s model γ′_c_. Higher values of albumin/globulin resulting from the lower globulin content in the serum correlate with lower values of γ′_c_, representing the shear rate at which red blood cell aggregation begins. As no signs of pathological aggregation (abnormal viscosity increase at lowest shear rates) were found in any of the blood samples, we speculate that the observed aggregation process refers to so-called rouleaux formation. The diameter of the capillaries is similar to the size of the erythrocyte. Blood flow in these conditions is made possible by the ability of red blood cells to deform in the flow [29,39]. Analysis of the correlation in Figure 7 suggests that the low values of γ′_c_ correspond to higher values of the albumin/globulin ratio, which, in turn, are associated with low values of globulins, including fibrinogen. The results obtained for the patients with silent cerebral ischemic foci show that a low value of γ′_c_ does not coexist with higher plasma fibrinogen and gamma globulin levels. In their study, Yoshiyasu et al. did not observe hyperfibrinogenemia in patients with silent cerebral ischemic foci [10].

Another correlation was found between Quemada’s model parameter *k_∞_* and plasma viscosity *η*_p_ (Figure 8). In both groups, the improved flexibility of erythrocytes, represented by small values of *k_∞_*, coexists with increased plasma viscosity. Both values are independent parameters, and both influence the viscosity of blood. Plasma viscosity depends on the composition of lipids and proteins [41,42,43]. At the same time, erythrocyte deformability is affected by several internal (inherent erythrocyte viscosity) and external factors (the surface area to volume ratio and the formability of the erythrocyte membrane) [44]. This study found a similar negative correlation between *k*_∞_ and *η*_p_ in patients and the control group (Figure 8). Improved flexibility of erythrocytes was demonstrated both in the test group and in the control, which indicates that in the test group, there was no increase in the rigidity of red blood cells.

A negative correlation between the parameter *k_∞_* characterizing the ability of red blood cells to deform and the fibrinogen level in both groups was found (Figure 9)—high levels of plasma fibrinogen correlate with reduced rigidity of red blood cells in both groups. The deformability of red blood cells is one of the conditions for blood flow in the microcirculation [2,3,26,27]. Improved deformability of erythrocytes and high plasma fibrinogen levels create conditions for the better alignment of erythrocytes in the packets, decreasing blood viscosity [9]. This mechanism may help to improve blood flow in the microcirculation.

The model parameter γ′_c_, specifying the value of the shear rate at which the process of creating packages starts, also correlates with the fibrinogen level (Figure 10). Both in the test and in the control group, a positive correlation was found. Other reports justify such a relationship, since the fibrinogen is actively involved in aggregating red blood cells. The bridges between erythrocytes, formed by fibrinogen molecules, bind packets and resist disaggregation forces induced by shear [36,45]. As similar correlations γ′_c_ (fibrinogen) were found (Figure 10) in both groups, one can conclude that higher values of γ′_c_, even in coexistence with hyperfibrinogenemia, do not play a role in the pathogenesis of creating ischemic lesions of the brain.

## 5. Conclusions

An increase in the fibrinogen level improves red cell elasticity and positively correlates with rouleaux formation.

Red cell deformability was not reduced in the group of patients with silent ischemic lesions of the brain. On the contrary, a better red cell elasticity in patients was observed. In connection with elevated plasma viscosity and fibrinogen levels, all of these features likely exemplify a self-regulatory mechanism maintaining an efficient blood flow in microcirculation. The increased content of high-molecular-weight plasma proteins does not induce silent ischemic lesions of the brain, but rather facilitates rouleaux formation to speed up the blood flow through the capillaries.

The results can be used to better understand the hemodynamics of blood flow and analyze the shear-dependent behavior of the aggregation and deformability of red blood cells. They may prove useful to evaluate the effect of various pharmacotherapeutic agents on the progression of the pathology, to identify new diagnostic indicators for screening, to perform risk assessments of cardiovascular disease and early diagnosis, as analytical tests for new therapeutic agents and to improve the understanding of patients’ response to drugs.

## Figures and Tables

**Table 1:** The demographic characteristics of the main group.

Main Group	Number of Patients	Mean Age	%	Median Age	Age Dispersion
Women	45	56 ± 3	65	55	25–78
Men	24	60 ± 3	35	61	42–76
Total	69	59 ± 3	100	60	25–78

**Table 2:** The demographic characteristics of the control group.

Control Group	Number of Patients	Mean Age	%	Median Age	Age Dispersion
Women	12	51 ± 4	71	54	25–77
Men	5	49 ± 4	29	52	25–76
Total	17	50 ± 4	100	54	25–77

**Table 3:** Summary of accompanying diseases for the study group and the control.

Diseases	Study Group	Control Group
Hypertension	26 persons	6
Atherosclerosis of anterior cerebral artery confirmed by Doppler ultrasonography	23 persons	2
Ischemic heart disease	14 persons	-
Diabetes mellitus	9 persons	1

**Table 4:** Values of rheological blood parameters in patients with clinically silent multifocal vascular cerebral lesions. Standard deviations of mean values are given as the error estimates.

Parameter	Control Group *n* = 17	Patients *n* = 69	*p*
Hct	41.4 ± 0.8	42.0 ± 0.4	-
Plasma viscosity *η*_p_ [mPa s]	1.44 ± 0.02	1.40 ± 0.01	-
*η* for *γ′* = 0.1 s^−1^ [mPa s]	26 ± 4	27 ± 2	-
*η* for *γ′* = 1 s^−1^ [mPa s]	13 ± 1	13.6 ± 0.6	-
*η* for *γ′* = 10 s^−1^ [mPa s]	5.5 ± 0.2	5.7 ± 0.1	-
*η* for *γ′* = 100 s^−1^ [mPa s]	3.24 ± 0.08	3.43 ± 0.07	-
*k*_0_	4.33 ± 0.11	4.20 ± 0.03	-
*k*_∞_	1.743 ± 0.048	1.637 ± 0.027	0.04
*γ*’_c_ [s^−1^]	5.7 ± 0.9	5.9 ± 0.5	-

**Table 5:** Values of biochemical blood parameters in patients with clinically silent multifocal vascular cerebral lesions. Standard deviations of mean values were given as the error estimates.

Parameter	Control Group *n* = 17	Patients *n* = 69	*p*
Fibrinogen [g/L]	4.2 ± 0.3	3.7 ± 0.1	0.047
IgM [g/L]	1.4 ± 0.1	0.99 ± 0.06	0.001
IgG [g/L]	9.2 ± 0.5	10.4 ± 0.3	0.05
IgA [g/L]	2 ± 1	2.3 ± 0.1	-
OB (ESR) [mm/h]	11 ± 2	11 ± 1	-
Albumin/globulin	1.8 ± 0.1	1.56 ± 0.03	0.015
Glucose [mmol/L]	5.24 ± 0.14	5.5 ± 0.1	-
Cholesterol [mmol/L]	5.52 ± 0.23	5.2 ± 0.1	-

## Data Availability

The data presented in this study are available on request from the corresponding author. The data are not publicly available due to privacy protection.
